# Translating prognostic quantification of c-MYC and BCL2 from tissue microarrays to whole slide images in diffuse large B-cell lymphoma using deep learning

**DOI:** 10.1186/s13000-023-01425-6

**Published:** 2024-01-19

**Authors:** Thomas E. Tavolara, M. Khalid Khan Niazi, Andrew L. Feldman, David L. Jaye, Christopher Flowers, Lee A.D. Cooper, Metin N. Gurcan

**Affiliations:** 1 Center for Artificial Intelligence Research, Wake Forest University School of Medicine, Winston-Salem, NC USA; 2 Present Address: Department of Laboratory Medicine and Pathology, Mayo Clinic, Rochester, MN USA; 3 Department of Pathology and Laboratory Medicine, Emory University School of Medicine, Atlanta, GA USA; 4 Department of Lymphoma/Myeloma, The University of Texas MD Anderson Cancer Center, Houston, TX USA; 5 Department of Pathology, Northwestern University Feinberg School of Medicine, Chicago, IL USA

**Keywords:** Deep learning, Diffuse large B-cell Lymphoma, c-MYC, BCL2, Immunohistochemistry, Multiple instance learning, Tissue microarrays, Whole slide images

## Abstract

**Background:**

c-MYC and BCL2 positivity are important prognostic factors for diffuse large B-cell lymphoma. However, manual quantification is subject to significant intra- and inter-observer variability. We developed an automated method for quantification in whole-slide images of tissue sections where manual quantification requires evaluating large areas of tissue with possibly heterogeneous staining. We train this method using annotations of tumor positivity in smaller tissue microarray cores where expression and staining are more homogeneous and then translate this model to whole-slide images.

**Methods:**

Our method applies a technique called attention-based multiple instance learning to regress the proportion of c-MYC-positive and BCL2-positive tumor cells from pathologist-scored tissue microarray cores. This technique does not require annotation of individual cell nuclei and is trained instead on core-level annotations of percent tumor positivity. We translate this model to scoring of whole-slide images by tessellating the slide into smaller core-sized tissue regions and calculating an aggregate score. Our method was trained on a public tissue microarray dataset from Stanford and applied to whole-slide images from a geographically diverse multi-center cohort produced by the Lymphoma Epidemiology of Outcomes study.

**Results:**

In tissue microarrays, the automated method had Pearson correlations of 0.843 and 0.919 with pathologist scores for c-MYC and BCL2, respectively. When utilizing standard clinical thresholds, the sensitivity/specificity of our method was 0.743 / 0.963 for c-MYC and 0.938 / 0.951 for BCL2. For double-expressors, sensitivity and specificity were 0.720 and 0.974. When translated to the external WSI dataset scored by two pathologists, Pearson correlation was 0.753 & 0.883 for c-MYC and 0.749 & 0.765 for BCL2, and sensitivity/specificity was 0.857/0.991 & 0.706/0.930 for c-MYC, 0.856/0.719 & 0.855/0.690 for BCL2, and 0.890/1.00 & 0.598/0.952 for double-expressors. Survival analysis demonstrates that for progression-free survival, model-predicted TMA scores significantly stratify double-expressors and non double-expressors (p = 0.0345), whereas pathologist scores do not (p = 0.128).

**Conclusions:**

We conclude that proportion of positive stains can be regressed using attention-based multiple instance learning, that these models generalize well to whole slide images, and that our models can provide non-inferior stratification of progression-free survival outcomes.

**Supplementary Information:**

The online version contains supplementary material available at 10.1186/s13000-023-01425-6.

## Background

Diffuse large B-cell lymphoma (DLBCL) is the most common subtype of non-Hodgkin lymphoma and accounts for around 40% of cases globally [1, 2]. In the United States alone, it has an estimated incidence of 24,500 annually [3]. According to 2016 and 2022 guidelines, the WHO recognizes several novel histopathological features and prognostic factors for DLBCL, including cell-of-origin classification (germinal center vs. activated B-cell), CD5 expression, and quantification of c-MYC and BCL2 expression in lymphoma cells as assessed by immunohistochemistry, referred to as double expressor status [4–7]. However, manual quantification of c-MYC and BCL2 can be subjective and may show intra- and inter-observer variability [8–10].

Recent studies have sought to develop automated methods to quantify immunohistochemical (IHC) markers through deep learning [11]. One of the earliest large studies predicted IHC scores and the proportion of positive stain from whole-slide IHC images [12]. Methods involved mapping hematoxylin and eosin (H&E) tumor regions onto IHC using adjacent tissue sections, training supervised convolutional neural networks (CNNs) on IHC patches, then applying some scheme to combine patch-level predictions. The best results achieved accuracies of around 90%. More recently, a similar study aimed to predict IHC scores from routine H&E whole-slide images (WSIs) [13]. Methods in this study resulted in areas under the curve (AUC) ranging from 0.50 to 0.84, similar to a previous study of ours [14]. Finally, one recent study utilized a commercially available software (Visiopharm) to detect positive and negative nuclei in c-MYC-stained whole slide images to predict the proportion of positive cells and achieved a Pearson correlation of 0.86 [8].

These and similar studies fail to exploit the key advantages of both tissue-imaging methods in the development of machine learning models for quantitative IHC. WSIs are more widely available but lead to greater inter- and intra- reader variability, given that pathologists must search for tumor-cell-rich areas on WSIs. This search process introduces variability, as pathologists may not select the same areas for analysis. Moreover, the process of selection is time-consuming due to the large size of WSIs. This feature makes WSIs less ideal for generating training data. On the other hand, tissue microarrays (TMAs), composed of multiple patient tissue samples, generally display much smaller tissue areas per case compared to most WSIs. This feature not only removes the variability in selecting an area for analysis (both in whole-slide digital or glass-slide reading) but also reduces the time it takes to find the said area. Thus, TMAs reduce inter- and intra- reader variability such that scores generated by manual microscopy as ground truth may be more reliable [15]. However, TMAs, which are expensive and time-consuming to construct, are not routinely used in clinical laboratories. By combining both tissue-preparation methods, we may exploit their advantages while mitigating their shortcomings.

**Fig. 1:**
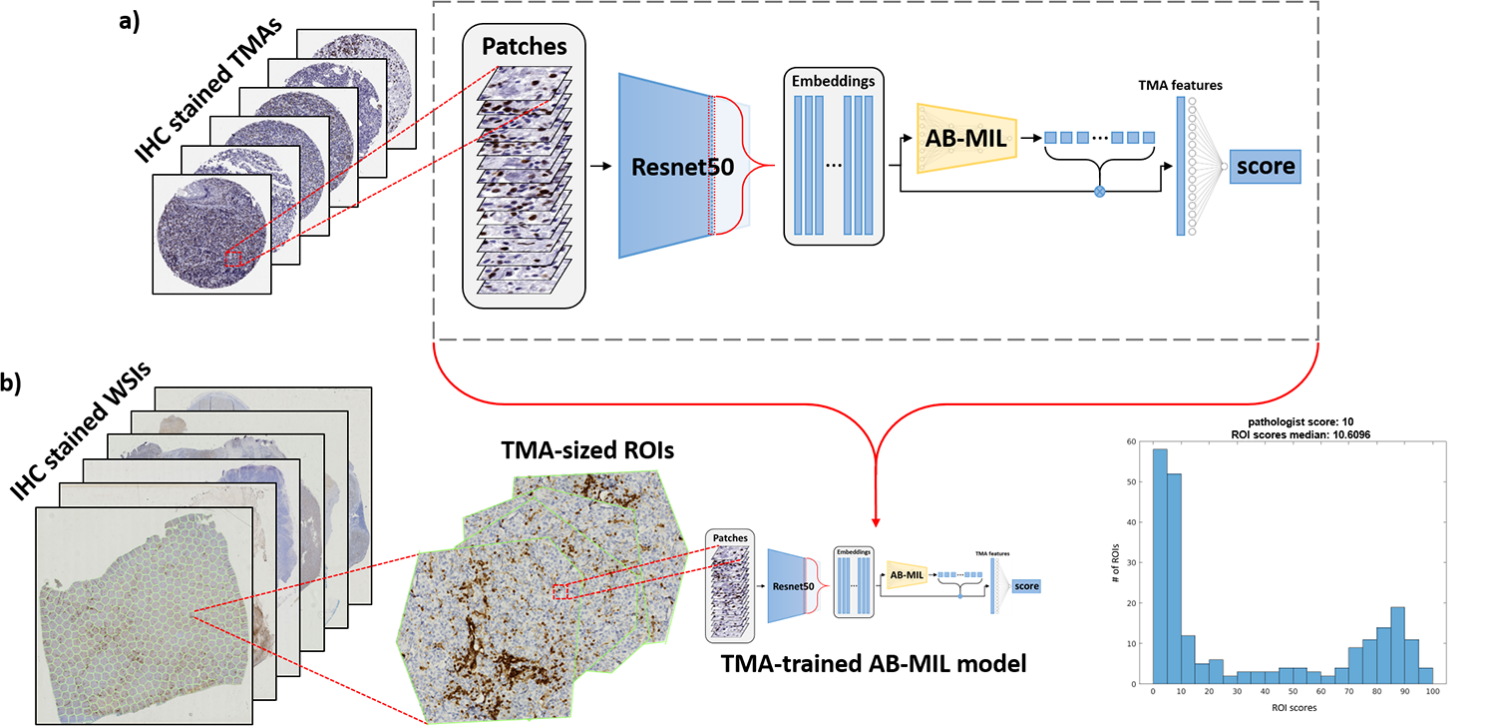
Overview of the proposed methodology. (**a**) AB-MIL is trained to predict c-MYC and BCL2 scores from TMAs. (**b**) Each WSI is decomposed into TMA-sized regions that are passed through the TMA-trained AB-MIL model. This generates a distribution of local WSI scores which are summarized using their median to predict the overall slide-level c-MYC or BCL2 score

We present a model that predicts the proportion of positive cells in c-MYC- and BCL2-stained tissue microarrays (TMAs). Unlike previous studies, our TMA-trained model can predict the proportion of positive tumor cells in c-MYC- and BCL2-stained WSIs. See Fig. 1 for an overview of the method. Because our method is trained on TMAs with limited and fixed search area, we expect the degree of inter-reader variability to be minimized. Thus, our method benefits from the accurate c-MYC and BCL2 scoring on TMAs. Furthermore, during inference on WSIs, our method automatically detects positive cell-rich regions, thus reducing the error associated with searching large WSIs. This novel strategy in our method achieves a high Pearson correlation on both TMAs and WSIs.

## Methods

### Datasets

For training we utilized the publicly available DLBCL-Morph dataset from Stanford consisting of digitized images of 378 TMA cores of DLBCL stained for c-MYC and BCL2 [1]. TMA slides were scanned at 40x objective magnification (0.25 μm per pixel) on an Aperio AT2 scanner (Leica Biosystems, Nussloch, Germany) in ScanScope Virtual Slide (SVS) format. Each TMA slide was prepared with a formalin-fixed, paraffin-embedded (FFPE) section of tumors assembled in a grid. Within the microarray each tumor is represented by a 0.6-mm core diameter sample in duplicate. Due to tissue-crush artifacts, some cores were removed from the study. The antibodies used for c-MYC and BCL2 were not disclosed in this study. In total, there were 173 patients with one or two cores each. Several pathologists determined the percentage of c-MYC- and BCL2-positive tumor cells in deciles that served as a continuous label for each patient case. Their names are not mentioned in the original paper [1]. Supplementary Fig. 1 summarizes the characteristics and utilization of this dataset.

For validation we utilized an external dataset consisting of 52 WSIs of DLBCL tissue sections stained for c-MYC and 56 WSI of DLBCL stained for BCL2. Two pathologists (David Jaye and Andrew L. Feldman, herein referred as “pathologist 1” and “pathologist 2”, respectively) determined the percentage of positive tumor cells for both stains in the decile that served as a continuous label for each patient. This external dataset came from the LEO study [16] and represented cases accrued from eight geographically dispersed institutions – Emory University (Atlanta, GA, USA), Cornell University (New York City, NY, USA), Grady Memorial Hospital (Atlanta, GA, USA), Iowa University (Iowa City, IA, USA), Mayo Clinic (Rochester, MN, USA), MD Anderson (Houston, TX, USA), University of Miami (Miami, FL, USA), and Washington University (St. Louis, MO, USA). The LEO dataset were also scanned on an Aperio AT2 scanner at 40X objective magnification (0.25 μm per pixel). These slides originate from the various labs of the LEO consortium and local hospitals in their vicinities. It is a real-world dataset that has considerable pre-analytic variability due to differences in tissue processing at the LEO partner sites. The LEO dataset can be accessed with permission at https://leocohort.org/contact-leo/. Supplementary Fig. 1 summarizes the characteristics and utilization of this dataset.

Only a subset of cases included imaging and c-MYC/BCL2 scores for both stains. As a result, in order to assess model performance on double-expressors, some cases were excluded. This resulted in a cohort of 171 patients for TMAs and 51 patients for WSIs.

### Attention-based multiple instance learning (AB-MIL) model

We applied an attention-based multiple instance learning (AB-MIL) [17] on patches with feature extracted using a ResNet50 model pre-trained using the ImageNet dataset. MIL is a machine learning paradigm where weak labels are assigned to collections of examples (called bags) rather than individual examples (called instances), like in conventional machine learning. MIL assumes that each instance has an implicit but unknown label. This presupposes that instances with certain labels are shared across all bags but that some bags possess some instances with different labels. Classification by MIL is therefore performed at the bag level and not the single instance level like in supervised learning.

**Fig. 2:**
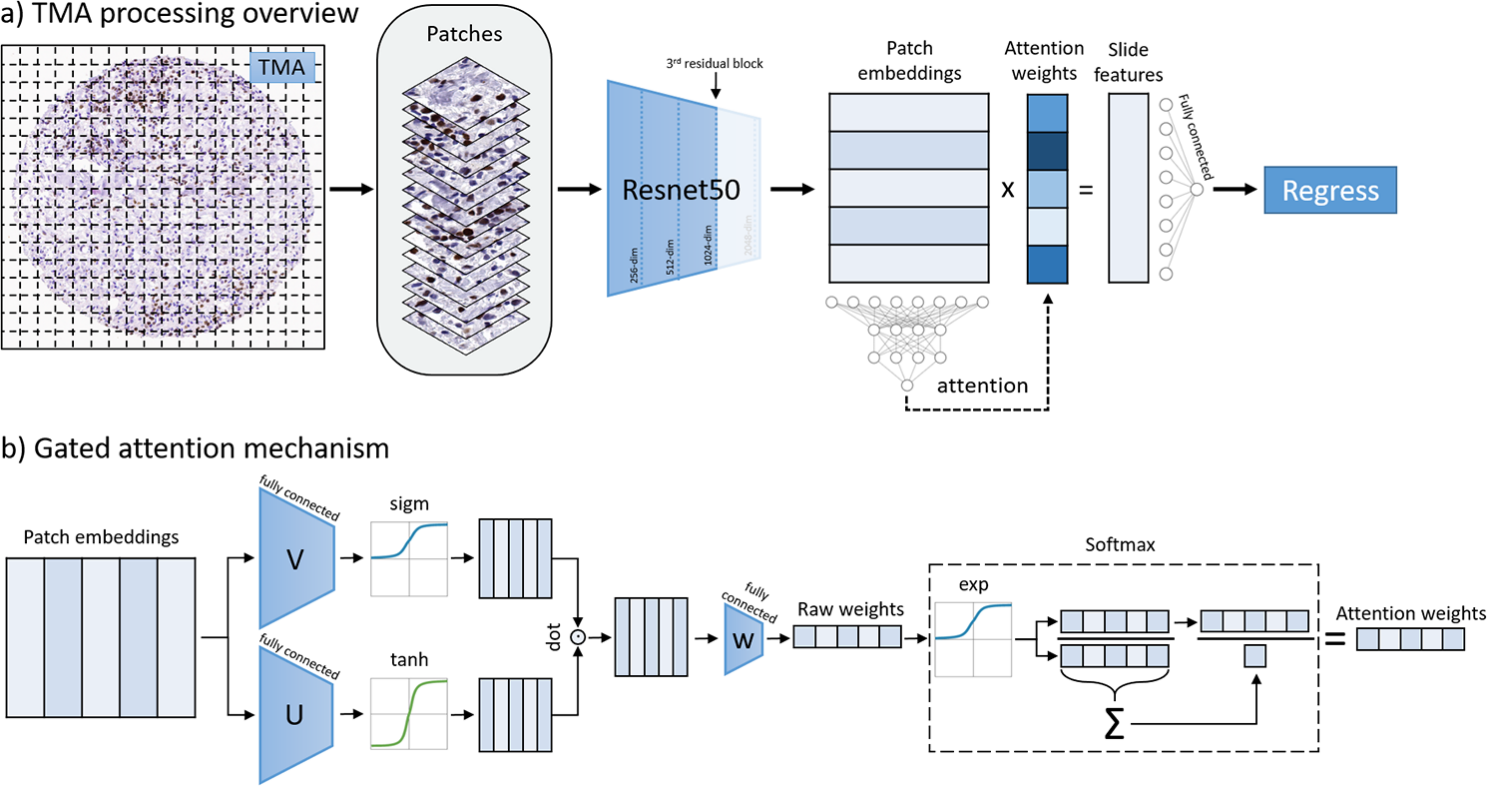
Overview of TMA processing and attention mechanism. (**a**) Each TMA is split up into small patches (instances). Each patch is passed through a pre-trained ResNet50. In different experimental settings, different levels of features are extracted from each patch. The first, second, third, and fourth residual blocks of ResNet50 yield 256-, 512-, 1024-, and 2048- dimensional embeddings, respectively, after spatial averaging. Each progressive block corresponds to more complex features. Finally, an AB-MIL is trained on these embeddings to regress the TMA c-MYC or BCL2 score. (**b**) The gated attention mechanism passes each embedding through parallel layers of the network (V and U) and is activated by tanh and sigmoid activation functions, respectively. The resulting parallel activations are dot-multiplied and passed through a final fully connected layer (w^T^), which maps the vector into a single value, its raw attention weight. These raw weights are scaled via softmax to weight attention weights

MIL relies on a method to aggregate the instances within a single bag. Though several methods exist, the *attention-based pooling* mechanism automatically learns to dynamically weight instances into a bag-level summary for calculating the regression [17]. For example, in our study a single TMA (bag), consists of many smaller image patches (instances). Feature are first extracted from each instance, forming instance embeddings. An *attention weight* is automatically computed for each embedded instance, and a weighted sum combines the instances into a core-level embedding. Regression is then performed on this core-level embedding. Figure 2a depicts this general process. In addition to equations (see Supplemental), we also pictorially depict the attention mechanism in Fig. 2b.

Patch-wise features were extracted using the first, second, third, and fourth residual blocks of a pretrained ResNet50 and individually spatially averaged to yield 256-, 512-, 1024-, and 2048- dimensional feature vectors for each 224 × 224 patch at 20x and 40x magnification, as in Fig. 2a. his emulates a popular process in the analysis of WSI by which each patch is represented by a feature vector [18, 19]. A fully-connected layer was prepended to the gated attention network to serve as a feature extractor for embedded patches. Furthermore, the output layer was modified to accommodate for regression (i.e., a single output) as in our previous work [14, 20]. Code is available at https://github.com/cialab/tma_to_wsi.

### Experimental approaches

We applied a ten-fold cross-validation with a split of 90/10 for training and testing on our TMA dataset. Preliminary experiments utilized other approaches (see Supplemental Materials). Following model cross-validation, we applied the trained models to our external testing set of WSI (see next section).

### Application to whole-slide images

**Fig. 3:**
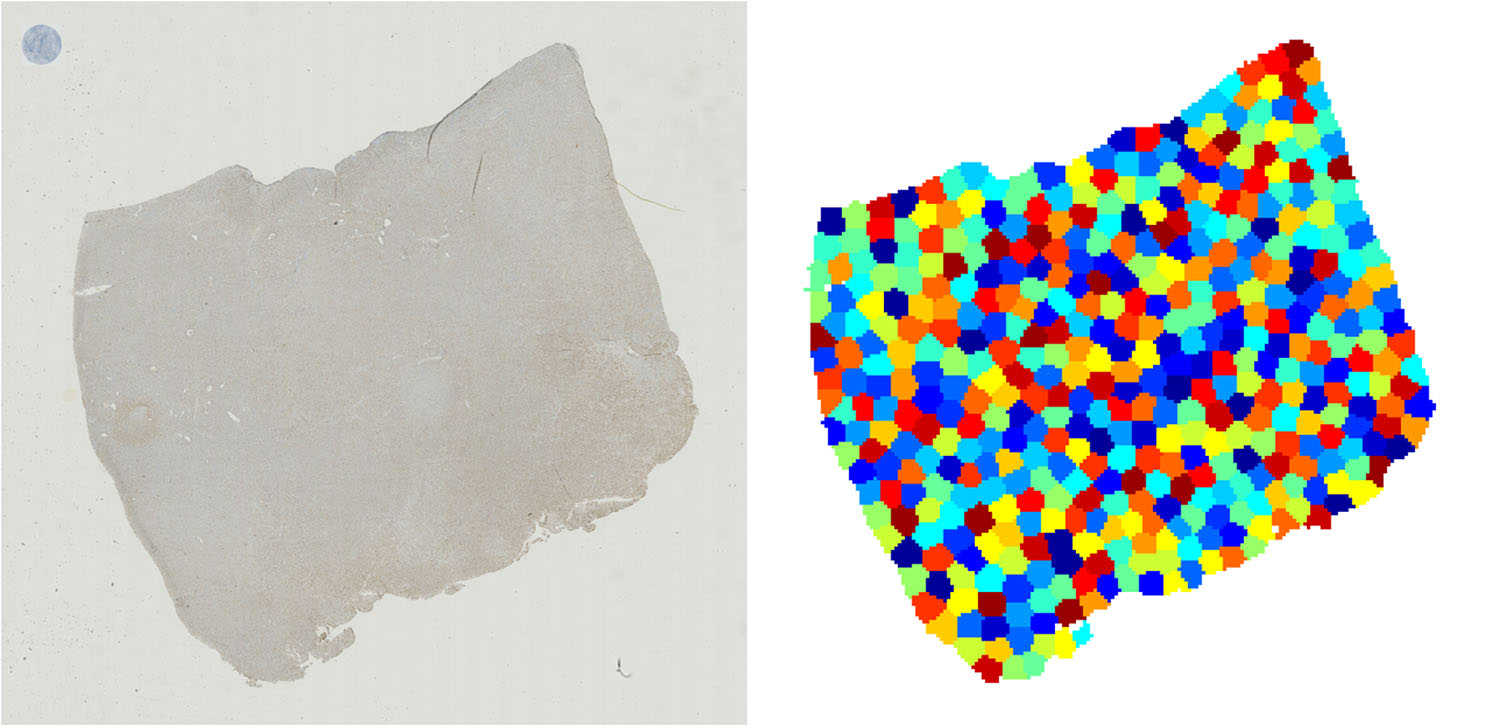
Coordinates in the WSI (left) are clustered to yield areas about the size of a TMA (right). A TMA with proportional size is given as an example on the top left of the WSI. Predictions are generated for each TMA-sized area then collapsed onto a single value using their median

Following experiments on TMAs, we utilized the models trained via our 10-fold cross-validation experiments to apply to WSIs. 224 × 224 patches were tiled at 20x magnification, and their features were extracted using the third residual block of ResNet50 as in TMAs. Coordinates of foreground patches were clustered using k-means clustering (see Fig. 3) to yield an average cluster size of 45 ± 7 patches with a range from 6 to 80. This cluster size coincides with the average number of non-overlapping patches obtained from TMAs in the previous step. K-means clustering based on coordinates was motivated by the fact that TMA-shaped regions (i.e., circles) extracted from WSIs would necessarily overlap. k-means clustering creates convex polygons which not only prevent this overlap but also approximate the shape of a circle. This resulted in several “mini-bags” for each WSI. These mini-bags were passed through respective pre-trained AB-MIL models to yield several predictions per WSI. The median of these predictions was taken to be the overall slide-level prediction.

### Statistical analysis

We utilized a number of statistical methods to assess the performance of our model. To evaluate the relationship between the predicted and actual c-MYC and BCL2 scores, we calculated the Pearson correlation coefficient. We used this metric to assess the strength and direction of the linear relationship between the predicted and actual c-MYC and BCL2 scores. Since it is sensitive to outliers, we also calculated the intragroup correlation coefficients (ICC) to assess the consistency of the model’s and pathologist’s predictions. We used a two-way random effects model with absolute agreement to calculate the ICC. We also calculated the sensitivity and specificity. Sensitivity measures the proportion of true positives correctly identified by the model, while specificity measures the proportion of true negatives correctly identified by the model. We used these metrics to evaluate the model’s ability to correctly classify positive and negative cases. Clinical thresholds of > 40% and > 50% for c-MYC and BCL2 were used to convert model and pathologists scores into positive and negative predictions [21]. Sensitivity and specificity for double-expressors was also computed using clinical thresholds. The Bland-Altman method was used to evaluate the agreement between the pathologist and model predictions. This method compares the differences between the pathologist and model predictions to their mean value. We calculated the limits of agreement as ± 1.96 standard deviations of the differences. Finally, survival analysis was used to evaluate the model’s and pathologist 1 and pathologist 2 abilities to predict time-to-event outcomes. Outcomes included overall survival and progression-free survival for TMAs as well as event-free survival for WSIs. High and low risk groups were defined by double-expressors and non double-expressors. Log-rank tests to compare the survival curves of different groups. All statistical analysis were carried out in MATLAB 9.4 with the exception of survival analyses, which were carried out in Python 3.8.5 with scikit-learn, pandas, numpy, scipy, and lifelines. 95% confidence intervals were computed for Pearson correlation, ICCs, sensitivities, and specificities using 1000-fold bootstrap with replacement.

## Results

### Prediction on tissue microarrays

Table 1 reports the results for automated c-MYC and BCL2 scoring on TMAs. In our preliminary experiments, the highest correlation, sensitivity, and specificity were achieved when utilizing patches extracted at 20x magnification and patch features extracted from the third residual block of ResNet50 (Supplementary Tables 1 and 2). All subsequent results were derived utilizing these parameters. AB-MIL finds a balance between sensitivity and specificity for c-MYC and BCL2 scoring relative to average pooling and is also more accurate for classifying double-expressors. Pearson correlation for c-MYC scoring is higher for AB-MIL, but for BCL2 scoring, average pooling higher. ICCs are similarly high for both c-MYC and BCL2 scoring on TMAs and are significant (p < 0.05). As with previously reported metrics, ICCs for attention pooling are higher than for average pooling (Supplementary Table 3). Wide confidence intervals for double-expressor sensitivity and specificity are likely due to the small number of double-expressors (n = 21). Bland-Altman plots indicate high agreement between model-generated scores and pathologist scores (Supplementary Fig. 2), with slight improvement from attention pooling. Additional results combining various experimental approaches to predict double-expressors are reported in Supplementary Table 4.

**Table 1:** Performance of AB-MIL c-MYC and BCL2 scoring as well as double-expressor performance on TMAs

Marker	Method	Pearson correlation	Sensitivity	Specificity
c-MYC	Baseline	0.842 [0.781,0.892]	0.596 [0.400,0.778]	0.993 [0.978,1.00]
	AB-MIL	0.862 [0.797,0.907]	0.702 [0.519,0.865]	0.966 [0.933,0.993]
BCL2	Baseline	0.928 [0.902,0.950]	0.862 [0.784,0.928]	0.950 [0.896,0.987]
	AB-MIL	0.905 [0.860,0.940]	0.885 [0.819,0.946]	0.949 [0.892,0.989]
Double-expressor	Baseline	-	0.44 [0.200,0.684]	1.00 [1.00,1.00]
	AB-MIL	-	0.711 [0.500,0.887]	0.974 [0.945,0.994]

Pearson correlation, sensitivity, and specificity are reported along with 95% confidence intervals in brackets. Average pooling is reported as a baseline comparison method

### Prediction on whole-slide images

Table 2 reports the Pearson correlation, sensitivity, and specificity between predicted c-MYC and BCL2 scores and pathologist 1 and pathologist 2 scores of WSIs. These results are based off attention-based TMA-trained models in Table 1. Overall, performance metrics for c-MYC remain relatively high, even slightly elevated. The opposite is true for BCL2, where there is a decline in performance across all metrics. Like in Table 1, the wide confidence intervals for double-expressors are likely due to the small number of double-expressors available (n = 8). ICCs show similar performance for both c-MYC and BCL2 scoring and are significant (Supplementary Table 3). Bland-Altman plots indicate moderate agreement between model-generated scores and pathologist scores, again with a decline relative to TMAs for BCL2 (Supplementary Fig. 2).

**Table 2:** Results of WSI c-MYC and BCL2 scoring from models trained with TMA data

Stain	Pearson correlation	Sensitivity	Specificity
c-MYC	0.883 [0.860,0.902] 0.753 [0.696,0.800]	0.857 [0.801,0.907] 0.706 [0.638,0.771]	0.991 [0.980,1.000] 0.930 [0.902,0.956]
BCL2	0.749 [0.703,0.790] 0.765 [0.728,0.798]	0.856 [0.820,0.891] 0.855 [0.816,0.890]	0.719 [0.654,0.779] 0.690 [0.628,0.751]
Double-expressor	-	0.890 [0.636,1.000] 0.598 [0.273,0.913]	1.000 [1.000,1.000] 0.952 [0.873,1.000]

Pathologist 1 and 2 are both used as references for Pearson correlation, sensitivity, and specificity metrics and are indicated by two respective metrics reported in each cell. Pearson correlation, sensitivity, and specificity are reported along with 95% confidence intervals in brackets

**Fig. 4:**
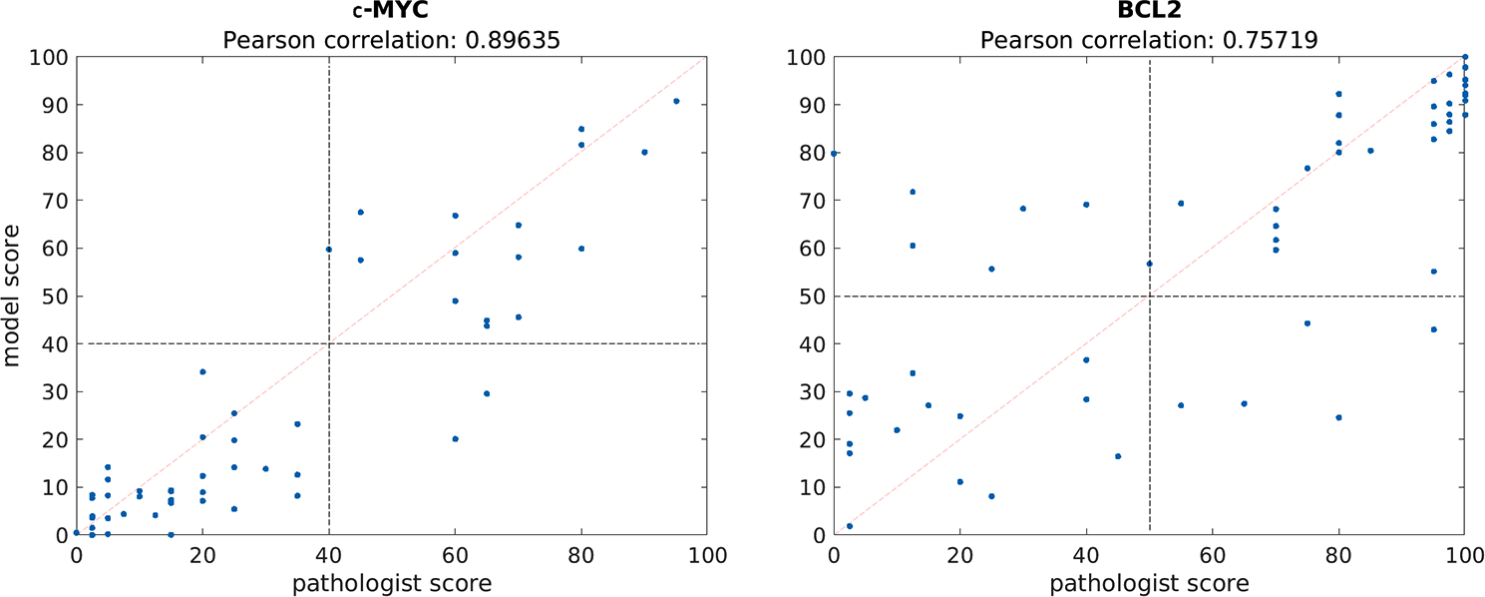
Pathologists’ scores are plotted against model-generated scores for c-MYC and BCL2 WSIs. The vertical and horizontal dotted lines represent clinical thresholds. Points in the top right and bottom left quadrant are true positives and true negatives, respectively. Likewise, points in the top left and bottom right are false positives and false negatives, respectively

Figure 4 depicts pathologist scores versus model prediction scores for WSIs. Visually, there is a positive trend for both stains. However, there are several extreme outliers for BCL2. For example, the model assigned as score of 80 to a TMA scored 0 by pathologist 1.

**Fig. 5:**
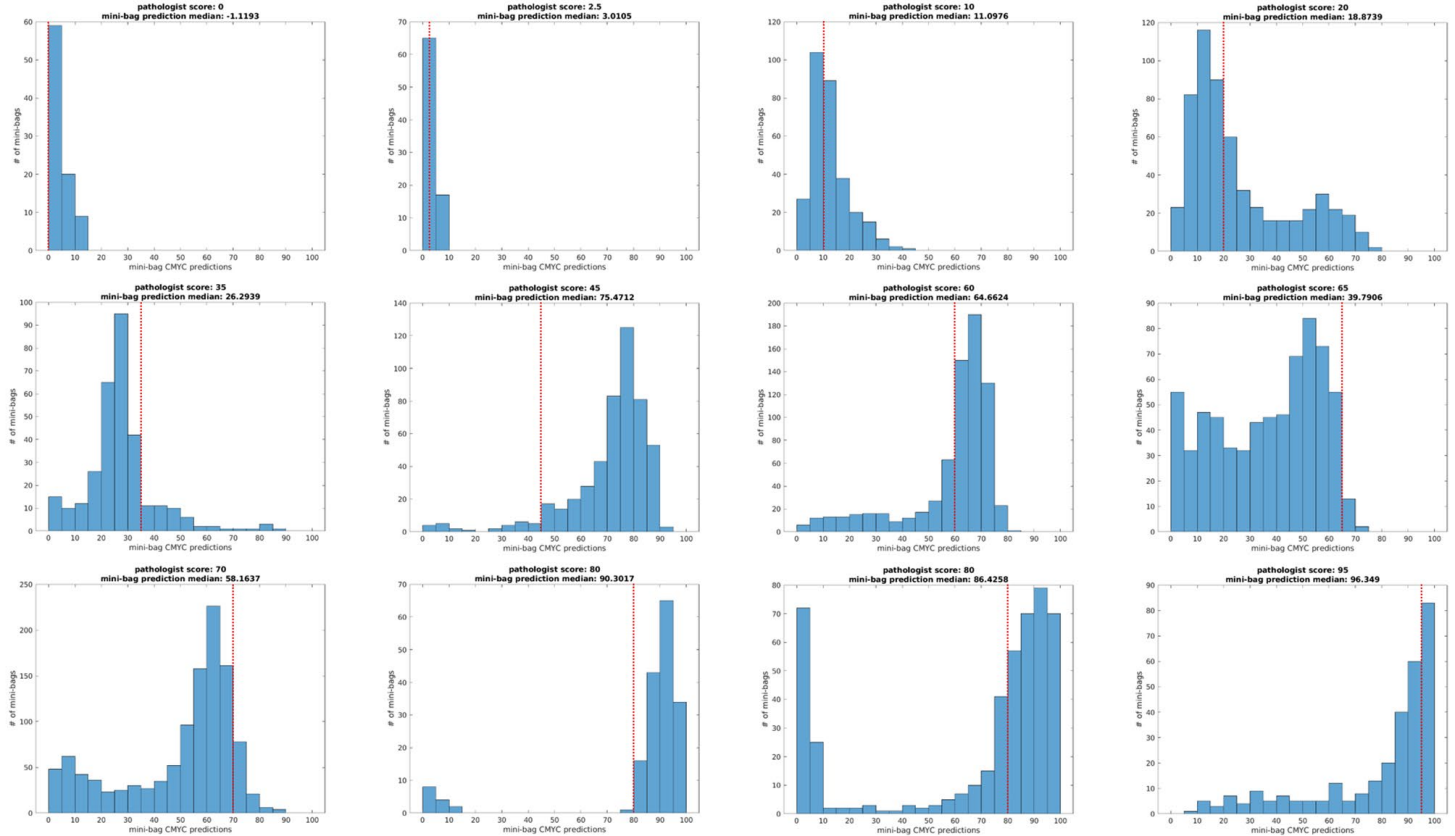
Distribution of c-MYC (six left) and BCL2 (six right) predictions for all mini-bags within a single WSI. A broad range of TMAs are presented along with pathologists’ and model-generated scores

Figure 5 depicts the distribution of predicted c-MYC scores from a few example slides. Distributions vary – some are bimodal, some are exponential, and some are normal. The varying distributions stem from the distribution of positive cells within WSIs; some areas are richer than others. However, it is in the areas that contain the highest density of cells that the overall slide-level label needs to be computed. This can be seen in Fig. 4, where it is clear that pathologist scores are near where the highest density of model-generated scores lies. The median seems to perform well, yet a summary statistic that captures this variation might improve results.

**Fig. 6:**
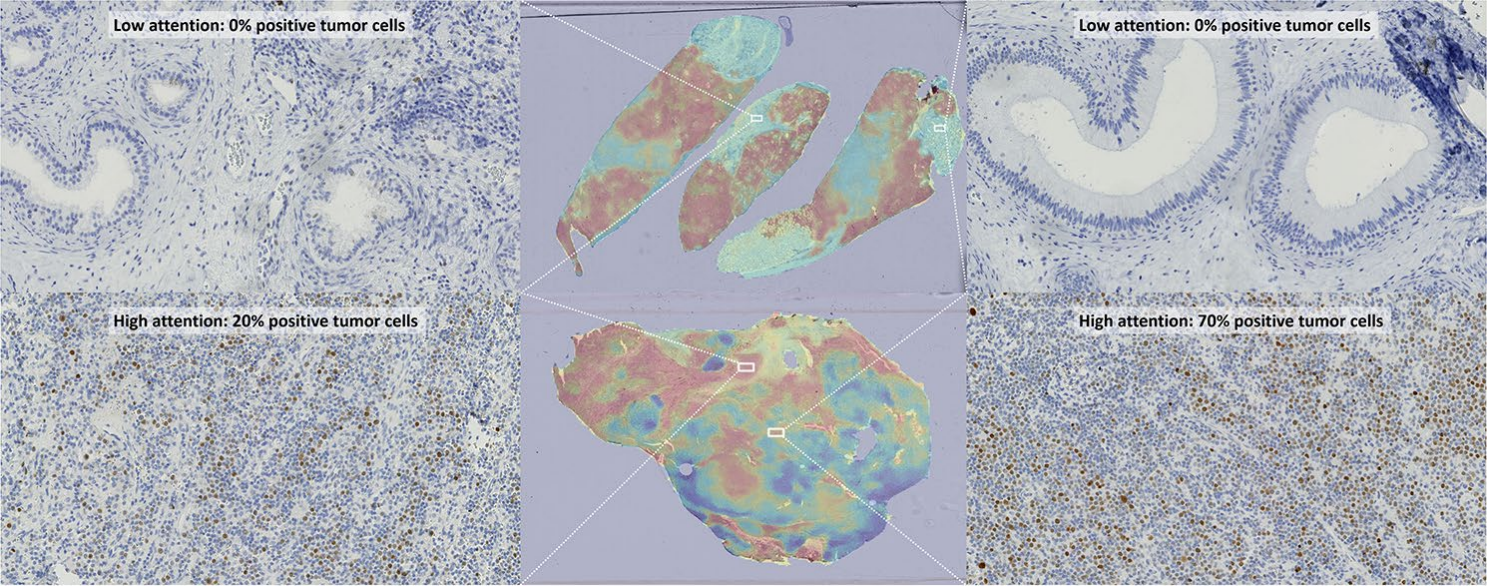
Attention heatmaps for our model on c-MYC WSIs. The model attends to tumor regions regardless of degree of positivity and does not attend to normal regions

Additionally, we generated attention heatmaps of our model on WSIs. One would expect to see the model attending to tumor regions of the tissue and ignoring normal areas. Furthermore, the model should attend to tumor regions regardless of degree of positivity. We can see that this is indeed the case in the examples in Fig. 6.

### Model scoring as a predictor for survival for double-expressors

**Fig. 7:**
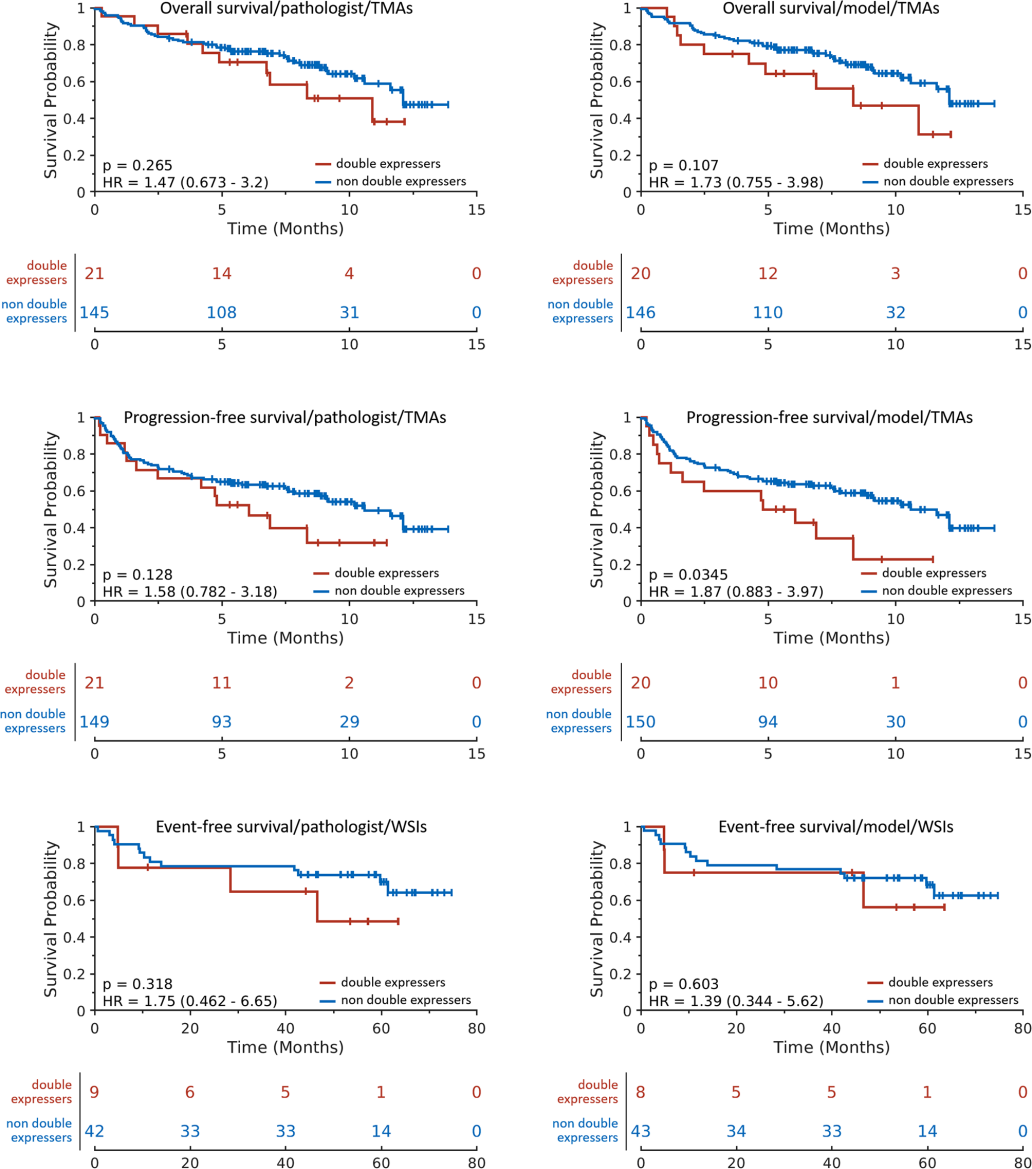
Survival curves for pathologist generated and model-predicted scores of TMAs and WSIs for double-expressors. Rows from top to bottom correspond to overall survival, progression-free survival, and event-free survival for TMAs, TMAs, and WSIs, respectively. The left and center columns corresponds to pathologist scoring, and the right column corresponds to model-generated scores. Overall, using standard clinical thresholds for c-MYC and BCL2, only one result is significant – model scoring for progression-free survival using TMAs

We performed additional analysis regarding the ability of model scores to predict survival for double-expressors (Fig. 7). We observed that for overall survival, both pathologist 1 and pathologist 2 generated and model-predicted TMA scores do not significantly stratify double-expressors and non double-expressors in terms of overall survival (p = 0.265 and p = 0.107, respectively). However, for progression-free survival, model-predicted TMA scores do significantly stratify double-expressors and non double-expressors (p = 0.0345), whereas pathologist generated scores do not (p = 0.128). As for WSIs, both pathologist 1 and pathologist 2 generated and model-predicted TMA scores do not significantly stratify double-expressors and non double-expressors in terms of event-free survival (p = 0.318 and p = 0.603, respectively).

**Fig. 8:**
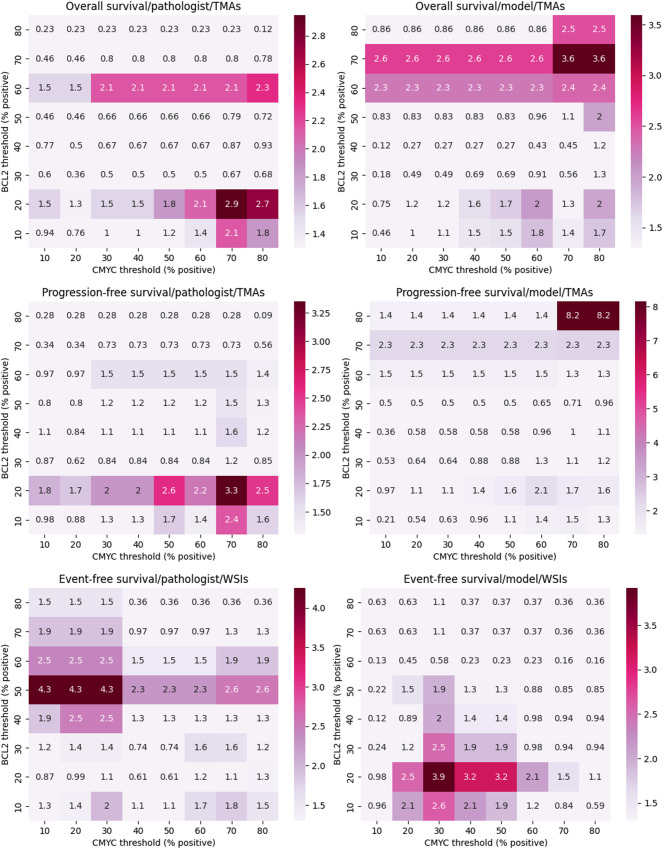
Log-rank analyses for pathologist generated and model-predicted scores of TMAs and WSIs using multiple c-MYC and BCL2. Each value represents the -log_10_(p-value) utilizing the specified thresholds for double-expressors. Any color indicates significance (i.e. >1.3). Rows and columns are arranged as in Fig. 7

When examining multiple thresholds for c-MYC and BCL2, we observe that patients are significantly stratified in terms of outcome risk at many combinations of thresholds (Fig. 8). In particular, we can see that for overall survival on TMAs, model-predicted scores stratify at more combinations of thresholds than pathologist scoring. However, for progression-free survival on TMAs event-free survival on WSIs, the opposite is observed.

## Discussion

In our current study, we utilized AB-MIL to predict the positive staining of IHC markers in tumor cells using regression. Previously, we have employed a similar model to predict HER2 scores from both H&E and HER2 [14] as well as for c-MYC TMAs [22]. The results of our current study report Pearson correlations for c-MYC and BCL2 that are comparable with similar studies predicting c-MYC-positivity [8].

Beyond simple application, we translate TMA-trained deep learning model to WSIs directly to predict IHC marker positivity. This serves as a proof of concept for c-MYC and BCL and other markers of interest, such as BCL6 in DLBCL. Additionally, we have also shown the potential of deriving WSI deep learning models from TMAs -- not just for IHC scoring. To the best of our knowledge, no other study has employed this approach before.

One advantage of the current study over similar studies [8] is that no manual threshold is required in order to segment positive and negative nuclei. It was reported in [8] that the commercially available software (Visiopharm) was initially utilized to segment tumor and non-tumor regions, background, areas of necrosis, and preparation artifacts. Then, a specific intensity threshold was selected to perform segmentation of positive and negative cells. Presumably, a stain separation was being performed in the background, and then a threshold was utilized for each stain channel to separate foreground from background. The proposed approach is not limited by the need for annotations as in Visiopharm. All that needs to be annotated is the overall c-MYC or BCL2 score for each TMA core or an equivalent sized region from a whole-slide image.

Moreover, the advantages of AB-MIL over traditional MIL approaches are clearly demonstrated throughout our results. One key advantage from an implementation perspective is the dynamic weighting offered by the method. Instead of pre-selecting a function (such as mean, max, or noisy-and [23]), AB-MIL can automatically learn a non-linear function to score importance of each instance, which then can be dynamically weighted into a slide-level feature representation in the same embedding space as the original instances. This advantage can be clearly seen in performance results in Tables 1 and 2. As a by-product, attention weights allow the model to be interpretable such that areas receiving high attention correspond to regions of the slide important to the overall slide-level label (in our case, c-MYC or BCL2 score). This clear advantage of interpretability can be seen in Fig. 6 – attended regions correspond to tumor regions. The utility of AB-MIL has already proven and continues to prove itself in several WSI regression and classification tasks [18, 20, 22, 24–31] but is facing healthy competition from more recent self-supervised, self-attention, and contrastive learning approaches [19, 32–34].

This study can be improved in several ways. Firstly, there are several weakly-supervised methods (some even based on AB-MIL) that perform classification of WSIs [14, 18, 20, 24, 25, 31]. Most are based on the same AB-MIL that we propose, but several methods utilize different approaches [32, 35]. These latter methods could be easily modified for regression as in AB-MIL and improve overall performance to produce additional comparisons. Second, our dataset was highly skewed towards scores between 10% and 40% for c-MYC. In fact, for scores of 70%, 80%, and 90%, there were only three, two, and three TMAs, respectively. Not only does this make it difficult to perform cross-validation (i.e., representation of each score in each training set), but it also biases the model to predict the most recurring values (i.e., those between 10% and 40%). Some of these errors are shown in Figs. 4 and 5. Thus, our method would benefit from additional data for these rare cases. Likewise, the number of double-expressors is quite small, and our analyses would benefit from inclusion of additional double-expressor samples. Third is the extent of followup for clinical outcome data. In our analyses, we only had access of overall survival and progression-free survival for TMAs as well as event-free survival for WSIs. Many of our subjects have censored outcomes because most do not require clinical follow-up. Fourth, given the intra- and inter-observer variability [8] for determining c-MYC and BCL2 positivity, the subjective nature of these scores represents “label noise”. This could potentially be improved by multiple readings or perhaps an alternative method for ground truth generation (i.e., molecular methods). Nonetheless, strong algorithms may emerge from noisy training labels [36]. Lastly, though the proposed algorithm can quantify positivity in both TMAs and WSIs as a whole, it cannot currently localize individual cells and classify their positivity. This would be a useful feature in clinical practice, as users would be able to verify why a certain count was made. However, such an approach requires training and validation with annotations of individual cell nuclei, thus nullifying the advantages granted by weak supervision and working with slide-level or TMA-level labels.

**Fig. 9:**
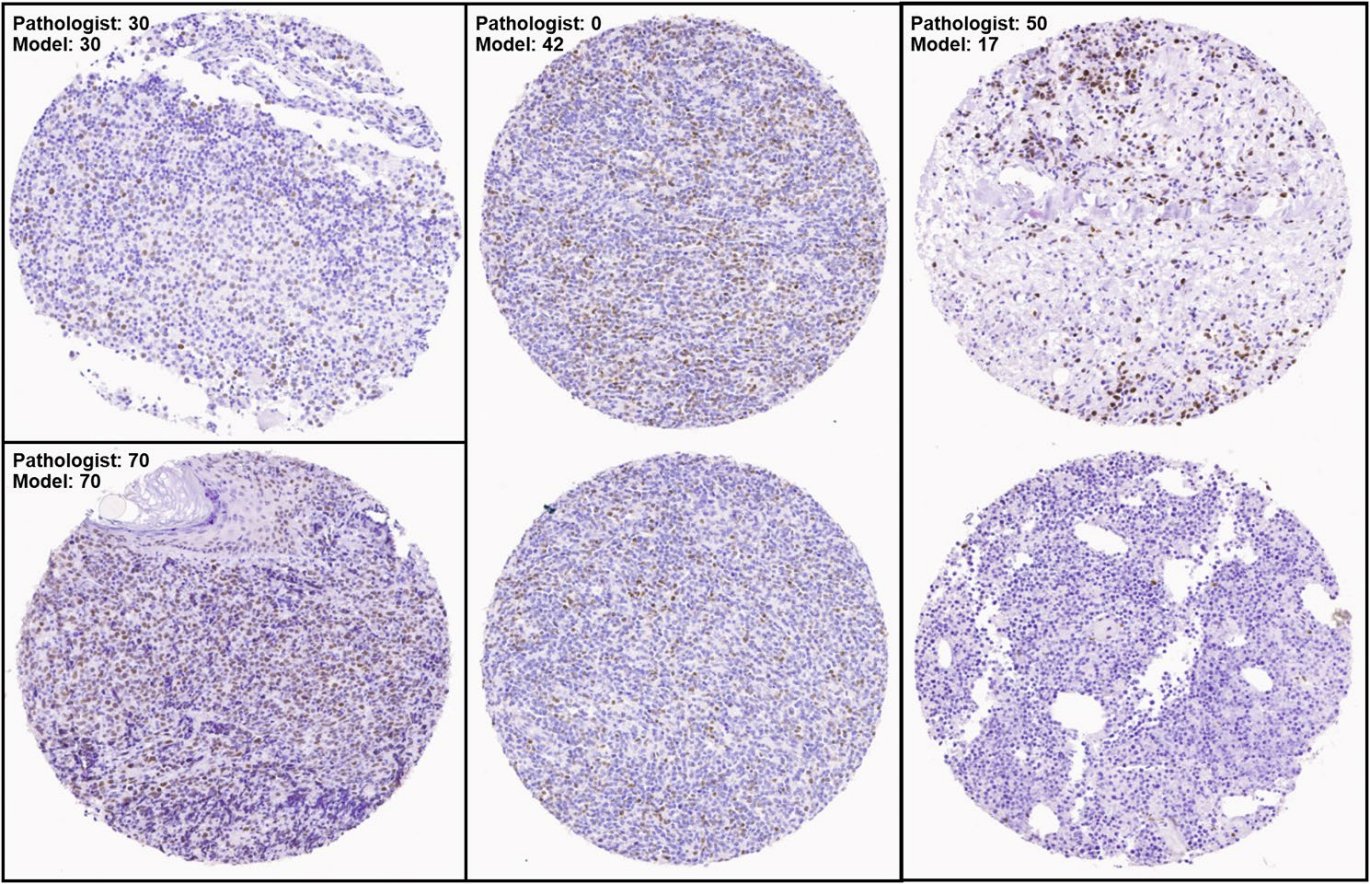
Examples of accurately regressed (left) and inaccurately (middle and right) regressed TMAs. The middle and right examples consisted of two TMAs with a single, unified score by the pathologist

Despite the high correlation between pathologist-scored TMAs and model-scored TMAs, there are instances in which the AB-MIL model fails. Examples are shown in Fig. 9, along with accurately regressed scores. There are two TMAs for which the model predicted the same exact proportions of positive tumor cells as the pathologist – 30% and 70%, respectively. However, there are two examples in which the model was not accurate – predicting 42% when the pathologist scored 0%, and predicting 17% when the pathologist scored 50%. For the former, it may be the case that the positively stained cells are lymphoid and that the model may have confused them for positive tumor cells. For the latter, there is a clear, dense cluster of positive tumor cells in the upper part of the top core. Perhaps it is the non-uniformity with which positive cells are distributed that confused the model.

**Fig. 10:**
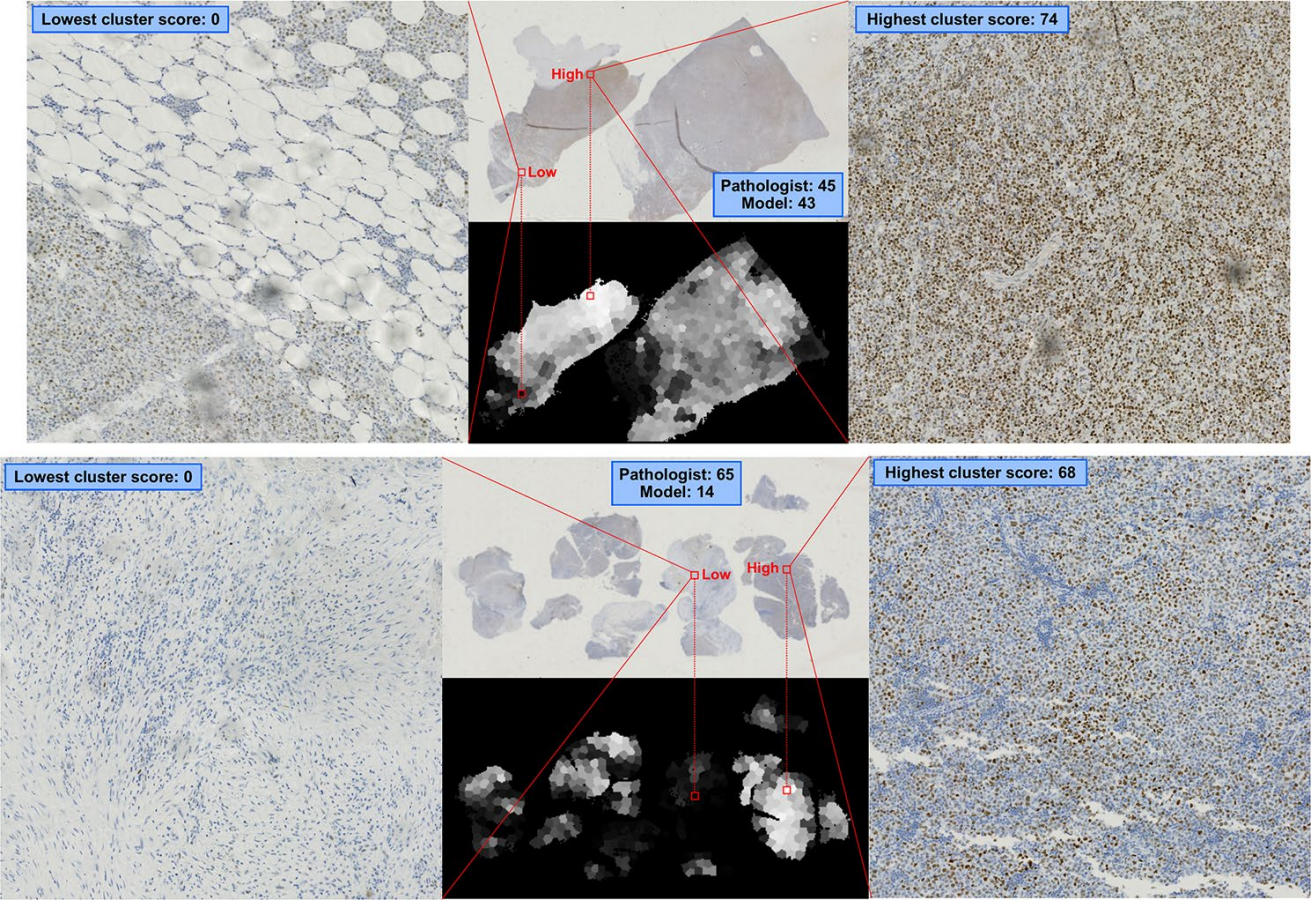
Examples of accurately (above) regressed and inaccurately (below) regressed WSIs. The grayscale images depict the scores given to each TMA-sized cluster (black = low, white = high). On the right and left are the lowest and highest clusters for each WSI

Figure 10 shows similar examples for WSIs. The top example depicts an accurately predicted WSI (pathologist 1: 45, model: 43), while the bottom depicts an inaccurately predicted WSI (pathologist 1: 65, model: 14). We can also see from grayscale images in Fig. 7 that depict scores for TMA-sized clusters. We automatically selected both the highest and lowest-scored clusters for each WSI. Clearly, the highest and lowest-scoring TMA-sized regions correspond to high and low cell positivity. Albeit not comprehensive, this suggests that the algorithm is able to detect the variation across each WSI.

## Conclusions

Here, we applied AB-MIL to predict the proportion of positive cells from c-MYC and BCL2 TMAs. Our method resulted in Pearson correlations of 0.8434 and 0.9188, respectively, depending on the cross-validation approach, along with a sensitivity and specificity of 0.7426 and 0.9627 when utilizing a clinical threshold of 40% for c-MYC and 0.9378 and 0.9509 when utilizing a clinical threshold of 50% for BCL2. For double-expressors, our model achieved a sensitivity and specificity of 0.7200 and 0.9736. We applied these trained models directly to WSIs and achieved a Pearson correlation of 0.8825 and 0.7485 for c-MYC and BCL2, respectively, along with a sensitivity and specificity of 0.8565 and 0.9911 for c-MYC, 0.8562 and 0.7186 for BCL2, and 0.8903 and 1.0000 for double-expressors. We also showed that for progression-free survival, model-predicted TMA scores significantly stratify double-expressors and non double-expressors (p = 0.0345), whereas pathologist generated scores do not (p = 0.128). We conclude that proportion of positive stains can be regressed using attention-based multiple instance learning and that these models translate well to whole slide images. Furthermore, our model significantly differentiates double expressor in terms of progression-free survival. Similar methods may be applied for the quantification of positive tumor cells. Although accurate, our method may be considered as a tool complementary to the pathologist’s workflow and may help in the reduction of pathologist’s workload. In future studies, we will evaluate the performance of our model on an external set of TMAs, other marker of interest (BCL6, CD10, and MUM1), and predict said markers directly from routine H&E. Finally, we would like to explore the predictive power of AB-MIL for other histological features of interest, such as ratio of positive c-MYC and BCL2 cells to total tissue area as well as ratio to total cells.

### Electronic supplementary material

Below is the link to the electronic supplementary material.

**Supplementary Material 1:** Additional methods and results

## Data Availability

TMAs are publicly accessible at https://wiki.cancerimagingarchive.net/pages/viewpage.action?pageId=119702520. The WSI dataset are available from https://leocohort.org/contact-leo/ on reasonable request. Code will be made available at https://github.com/cialab/tma_to_wsi.

## Abbreviations

AB-MIL: Attention-based multiple instance learning
DLBCL: Diffuse large B-cell lymphoma
ICC: Intragroup correlation coefficients
IHC: Immunohistochemistry
LEO: Lymphoma epidemiology of outcomes
TMA: Tissue microarray
WSI: Whole-slide image
