# Supplementary material for: Translating prognostic quantification of c-MYC and BCL2 from tissue microarrays to whole slide images in diffuse large B-cell lymphoma using deep learning
Source: Diagn Pathol. 2024 Jan 19;19:17. doi: 10.1186/s13000-023-01425-6 (PMC10797911; doi:10.1186/s13000-023-01425-6)
Supplement: Supplementary file 1 — Supplementary Material 1: Additional methods and results [file 13000_2023_1425_MOESM1_ESM.docx]

**Methods**

AB-MIL

The *gated attention* mechanism consists of two parallel, fully connected networks. Each instance embedding (h_k_) is passed through both parallel layers of the network (V and U) and is activated by tanh and sigmoid activation functions, respectively. The resulting parallel activations are dot-multiplied and passed through a final fully connected layer (w^T^), which maps the vector into a single value, its attention weight (a_k_). Equation 2 summarizes these interactions. The weighted sum of each embedded instance and its attention weight yields a bag-level instance (z), as in Equation 1. The parameters (V,U,w) for this neural network are automatically learned through the training of the model [17].

| $z=\sum_{k=1}^{K} a_{k}h_{k}$ | 1 |
| --- | --- |
| $a_{k}=\frac{exp\left\{ w^{T}tanh\left( Uh_{k}^{T} \right)\cdot w^{T}sigm\left( Vh_{k}^{T} \right) \right\}}{\sum_{j=1}^{K} exp\left\{ w^{T}tanh\left( Uh_{k}^{T} \right)\cdot w^{T}sigm\left( Vh_{j}^{T} \right) \right\}}$ | 2 |

Experimental approaches

Models were trained using Adam for at least 100 epochs with a learning rate of 0.0003, weight decay of 0.005, betas of 0.9/0.99, and mean-squared error loss. Training was halted if validation loss did not decrease for 20 consecutive epochs.

We adopted three cross-validation approaches to determine the generalization of the proposed method. First, we applied a 100-fold Monte-Carlo cross-validation with a training/validation/testing split of 80/10/10. Based on these results, we determined an optimal magnification and feature space. Second, we applied leave-one-out cross-validation and ten-fold cross-validation with a split of 90/10 for training and testing.

**Results**

Prediction from TMAs

Supplementary Table 1 reports the results of additional experimental approaches for CMYC on TMAs. For 100-fold Monte-Carlo, the highest correlation was achieved using 20x features from the third Resnet50 residual block (0.8421+/-0.1268). This exceeded that of other residual blocks as well as 40x magnification. For the leave-one-out and 10-fold experiments, the resulting correlations were 0.8648 and 0.8605, respectively.

| Supplementary Table 1. Results of each experimental approach for CMYC scoring. Pearson correlation, sensitivity, and specificity are reported along with 95% confidence intervals in brackets. | | | | | |
| --- | --- | --- | --- | --- | --- |
| **Experiment** | **Scale** | **Residual block (dim)** | **Pearson correlation** | **Sensitivity** | **Specificity** |
| 100-fold Monte-Carlo | 20x | 1 (d=256) | 0.0475  [-0.0061,0.1010] | 0.0000 [0.0000,0.0000] | 1.0000 [1.0000,1.0000] |
|  | 20x | 2 (d=512) | 0.5575 [0.5177,0.5979] | 0.2425 [0.1917,0.2957] | 0.9810 [0.9738,0.9875] |
|  | 20x | 3 (d=1024) | 0.8530 [0.8341,0.8699] | 0.7426 [0.6877,0.7952] | 0.9627 [0.9523,0.9727] |
|  | 20x | 4 (d=2048) | 0.8434 [0.8229,0.8632] | 0.6722 [0.6160,0.7294] | 0.9612 [0.9513,0.9706] |
|  | 40x | 3 (d=1024) | 0.8151 [0.7920,0.8377] | 0.6756 [0.6203,0.7327] | 0.9584 [0.9470,0.9688] |
| Leave-one-out | 20x | 3 (d=1024) | 0.8599 [0.7994,0.9054] | 0.7088 [0.5323,0.8667] | 0.9583 [0.9244,0.9866] |
| 10-fold CV | 20x | 3 (d=1024) | 0.8620 [0.7971,0.9066] | 0.7018 [0.5186,0.8652] | 0.9656 [0.9329,0.9930] |
|  | 40x | 3 (d=1024) | 0.8440 [0.7802,0.8946] | 0.7376 [0.5714,0.9091] | 0.9598 [0.9276,0.9868] |

Supplementary Table 2 reports the results of each of our experimental approaches for BCL2 on TMAs. For 100-fold Monte-Carlo, the highest correlation was achieved using 40x features from the third Resnet50 residual block (0.9169+/-0.0593). This exceeded the performance of 20x magnification. For the leave-one-out and 10-fold experiments, the resulting correlations were 0.9159 and 0.9182 at 40x magnification, respectively, both exceeding 20x performance.

| Supplementary Table 2. Results of each experimental approach for BCL2 scoring. Pearson correlation, sensitivity, and specificity are reported along with 95% confidence intervals in brackets. | | | | | |
| --- | --- | --- | --- | --- | --- |
| **Experiment** | **Scale** | **Residual block (dim)** | **Pearson correlation** | **Sensitivity** | **Specificity** |
| 100-fold Monte-Carlo | 20x | 3 (d=1024) | 0.9043 [0.8920,0.9150] | 0.9029 [0.8843,0.9215] | 0.9400 [0.9246,0.9556] |
|  | 40x | 3 (d=1024) | 0.9137 [0.9023,0.9247] | 0.9113 [0.8934,0.9295] | 0.9399 [0.9220,0.9573] |
| Leave-one-out | 20x | 3 (d=1024) | 0.9156 [0.8750,0.9454] | 0.9257 [0.8681,0.9733] | 0.9504 [0.8976,0.9880] |
|  | 40x | 3 (d=1024) | 0.9188 [0.8786,0.9509] | 0.9378 [0.8867,0.9802] | 0.9509 [0.8982,0.9885] |
| 10-fold CV | 20x | 3 (d=1024) | 0.9045 [0.8603,0.9397] | 0.8847 [0.8191,0.9459] | 0.9486 [0.8924,0.9886] |
|  | 40x | 3 (d=1024) | 0.9157 [0.8784,0.9463] | 0.9150 [0.8556,0.9667] | 0.9379 [0.8782,0.9872] |

Supplementary Table 3 reports the intragroup correlation coefficients (ICC) for both TMAs and WSIs. Means along with 95% confidence intervals are reported.

| Supplementary Table 3. ICC for both TMAs and WSIs. Means along with 95% confidence intervals are reported. All p-values are less than 0.05. | | | | | | | |
| --- | --- | --- | --- | --- | --- | --- | --- |
| Stain | Pooling (medium) | 1-1 | 1-k | C-1 | C-k | A-1 | A-k |
| CMYC | Mean (TMA) | 0.8275 [0.7527,0.8856] | 0.9053 [0.8589,0.9393] | 0.8278 [0.7533,0.8856] | 0.9054 [0.8593,0.9394] | 0.8276 [0.7528,0.8856] | 0.9053 [0.8590,0.9393] |
|  | Attention (TMA) | 0.8540 [0.7890,0.9039] | 0.9210 [0.8821,0.9495] | 0.8540 [0.7885,0.9036] | 0.9210 [0.8818,0.9494] | 0.8540 [0.7890,0.9039] | 0.9210 [0.8820,0.9495] |
|  | Attention (WSI) | 0.8612 [0.8334,0.8847] | 0.9254 [0.9091,0.9388] | 0.8821 [0.8598,0.9016] | 0.9373 [0.9246,0.9483] | 0.8628 [0.8355,0.8857] | 0.9263 [0.9104,0.9394] |
| BCL2 | Mean (TMA) | 0.9254 [0.8973,0.9469] | 0.9612 [0.9459,0.9727] | 0.9254 [0.8977,0.9469] | 0.9612 [0.9461,0.9727] | 0.9254 [0.8974,0.9469] | 0.9612 [0.9459,0.9727] |
|  | Attention (TMA) | 0.8962 [0.8560,0.9309] | 0.9452 [0.9224,0.9642] | 0.8967 [0.8566,0.9317] | 0.9454 [0.9228,0.9647] | 0.8963 [0.8560,0.9309] | 0.9452 [0.9224,0.9642] |
|  | Attention (WSI) | 0.7351 [0.6913,0.7768] | 0.8471 [0.8175,0.8744] | 0.7351 [0.6916,0.7767] | 0.8471 [0.8177,0.8743] | 0.7351 [0.6912,0.7768] | 0.8471 [0.8174,0.8744] |

Supplementary Table 4 reports the result of combining the results of CMYC and BCL2 scoring to predict double expressers. Regardless of which experimental approaches and scales are combined, sensitivity and specificity remain around 0.71 and 0.96, respectively. Wide confidence intervals are likely due to the small number of double expressers (n=21).

| Supplementary Table 4. Combining results of CMYC and BCL2 scoring to predict double expressers. Sensitivity and specificity are reported along with 95% confidence intervals. | | | |
| --- | --- | --- | --- |
| **CMYC experiment/scale** | **BCL2 experiment/scale** | **Sensitivity** | **Specificity** |
| Leave-one-out/20x | Leave-one-out/20x | 0.7133 [0.5000,0.9000] | 0.9665 [0.9373,0.9931] |
| Leave-one-out/20x | Leave-one-out/40x | 0.7155 [0.5000,0.8918] | 0.9666 [0.9338,0.9933] |
| Leave-one-out/20x | 10-fold/20x | 0.7083 [0.5000,0.8918] | 0.9669 [0.9346,0.9932] |
| Leave-one-out/20x | 10-fold/40x | 0.7080 [0.5000,0.9048] | 0.9664 [0.9322,0.9933] |
| 10-fold/20x | Leave-one-out/20x | 0.7103 [0.5200,0.9167] | 0.9735 [0.9465,0.9935] |
| 10-fold/20x | Leave-one-out/40x | 0.7167 [0.5000,0.9077] | 0.9739 [0.9461,0.9936] |
| 10-fold/20x | 10-fold/20x | 0.7114 [0.5000,0.8868] | 0.9735 [0.9446,0.9935] |
| 10-fold/20x | 10-fold/40x | 0.7200 [0.5217,0.9130] | 0.9736 [0.9446,0.9935] |

| 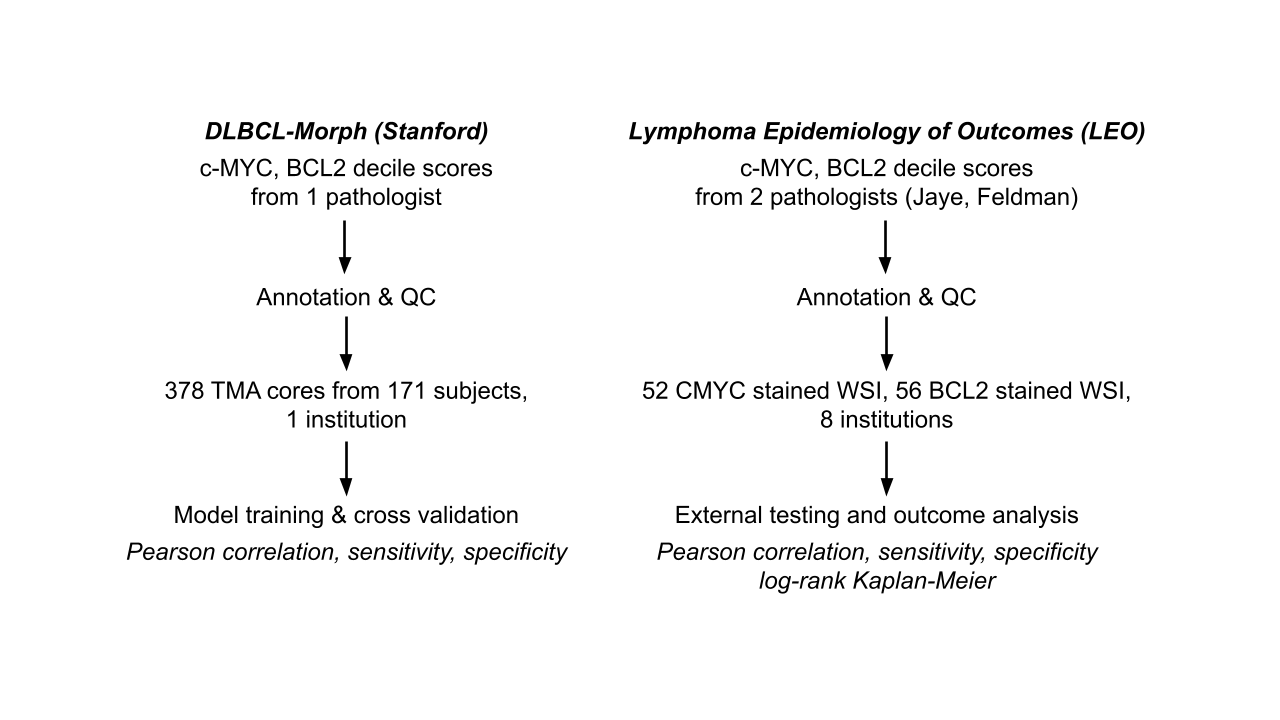 |
| --- |
| Supplementary Figure 1. Overview and summary of the TMA dataset (left) and WSI dataset (right). The TMA dataset is used for training and cross validation. The WSI dataset is used for external testing and outcome analysis. |

| 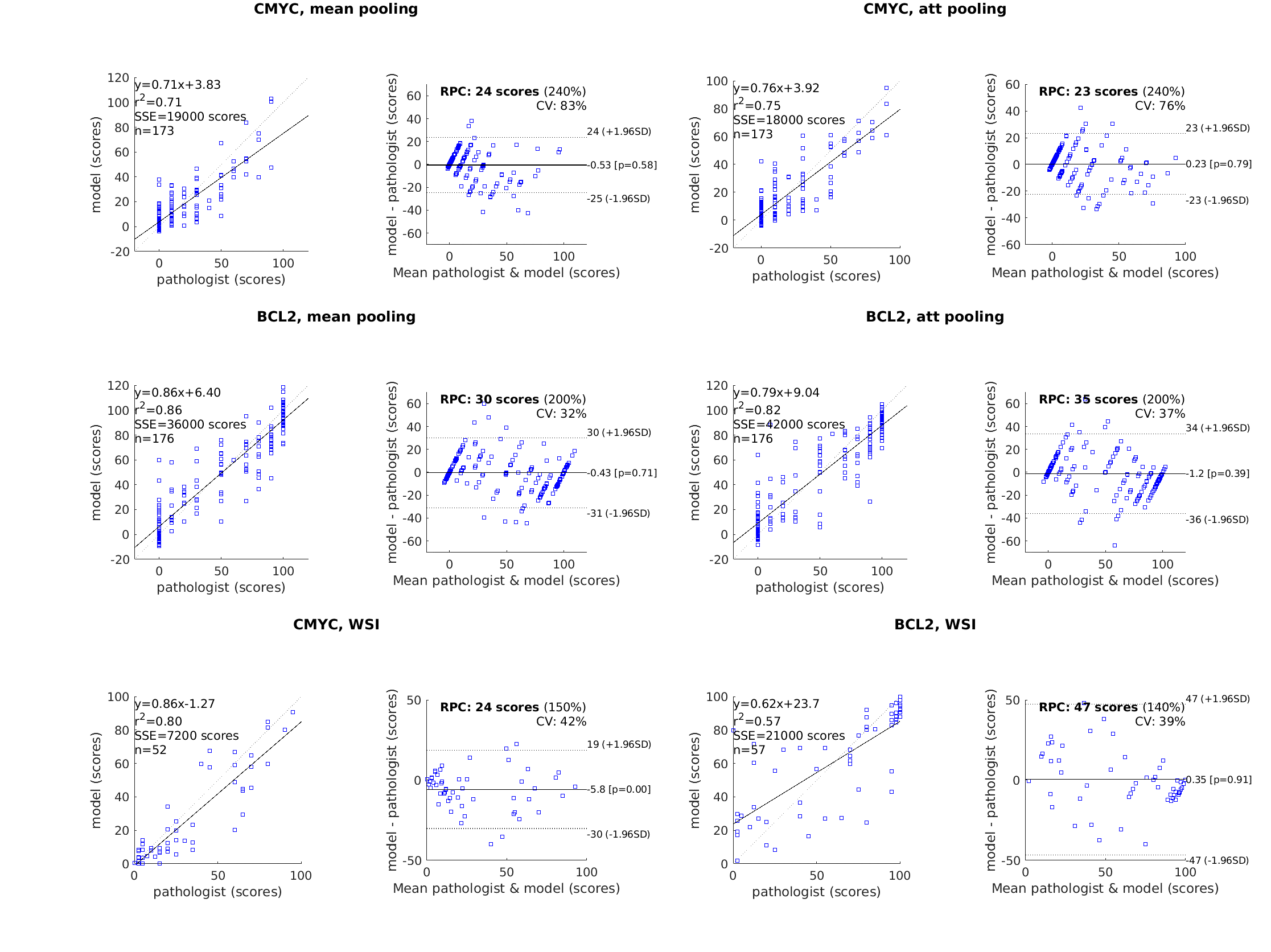 |
| --- |
| Supplementary Figure 2. Bland-Altman plots to evaluate the agreement between the pathologist and model predictions. We calculated the limits of agreement as ±1.96 standard deviations of the differences. Results are shown for mean pooling and attention pooling for TMAs as well as for WSIs. |

| 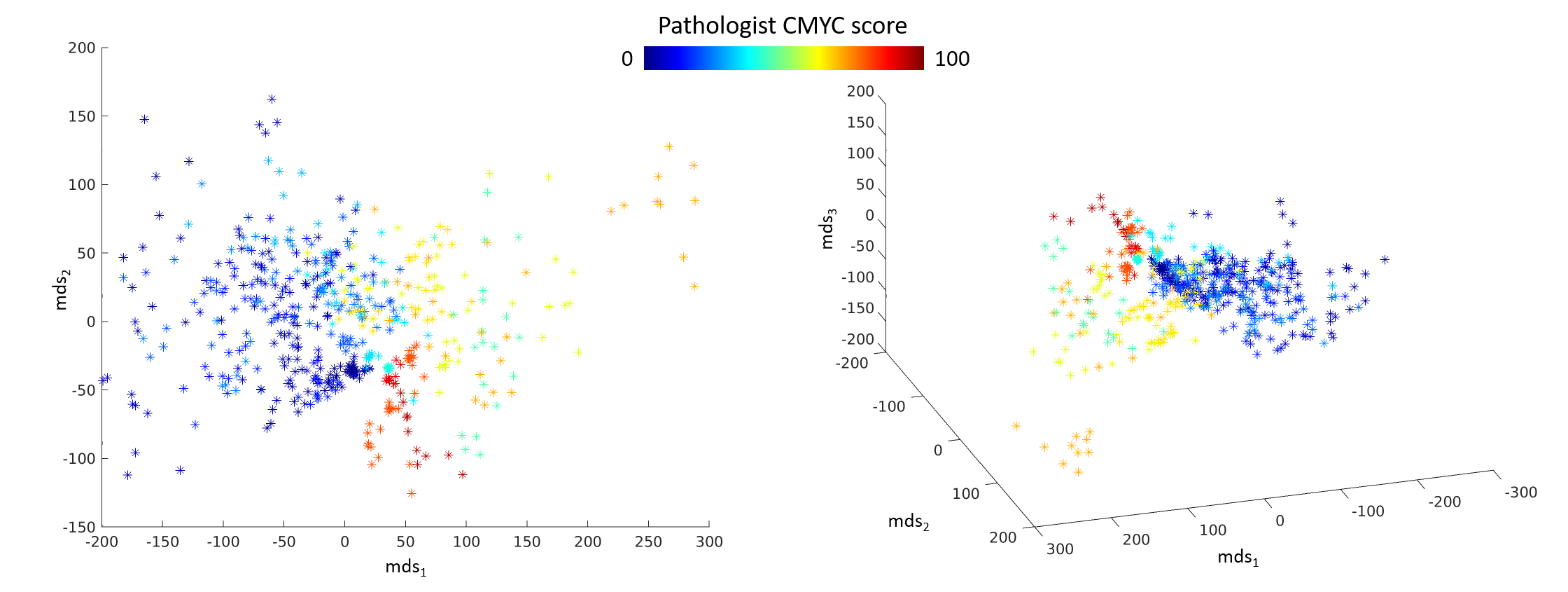 |
| --- |
| 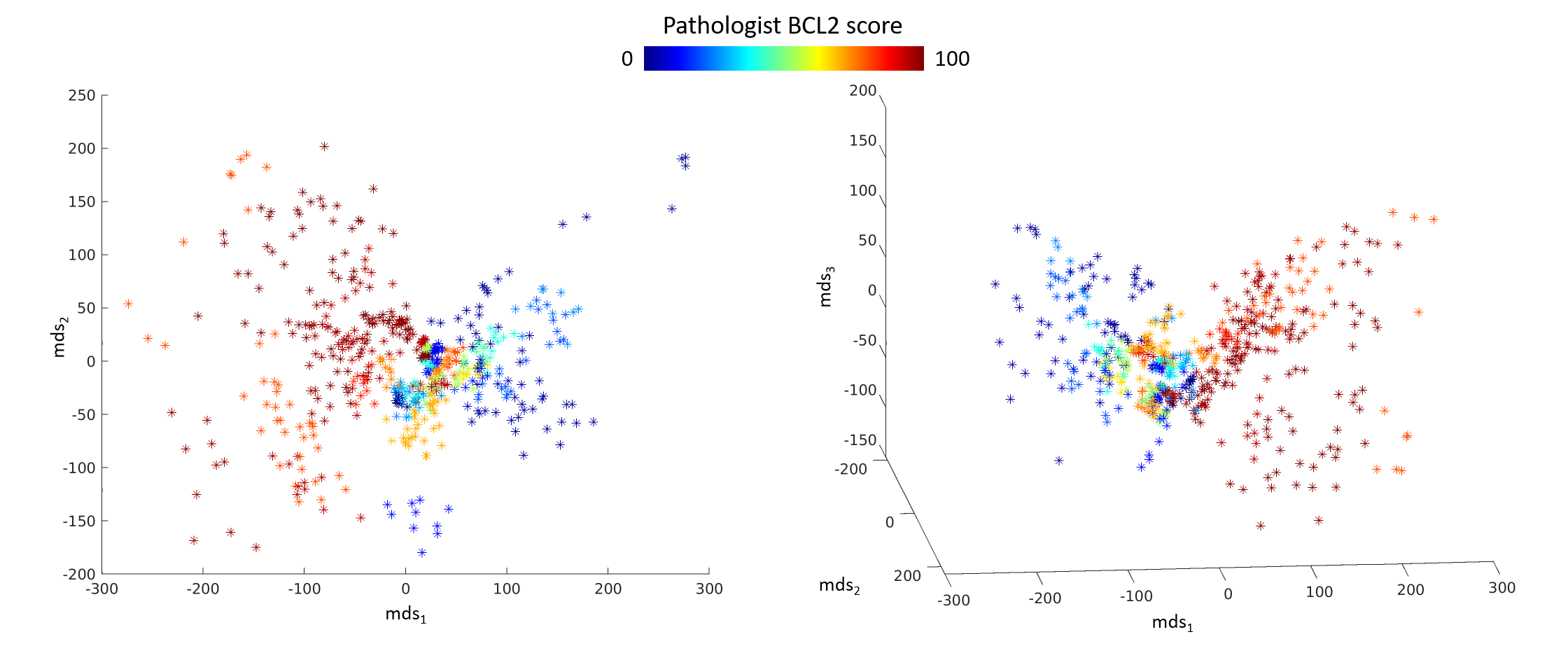 |
| Supplementary Figure 3. Multi-dimensional scaling projections of CMYC and BCL2 slide-level representations obtained by attention MIL on WSIs – 2-dimensional on the left and 3-dimensional on the right. A jet color map is used to indicate the ground truth positivity score – red is 100, and blue is 0. Clearly, slide-level representations cluster according to their respective positivity score. mds_x_ dimensions refer to a low dimensional representation of the slide-level feature space the preserves the distances between each data point. |
